# Isolated Right Subclavian Artery From the Pulmonary Artery in D-Transposition of the Great Arteries

**DOI:** 10.1016/j.case.2022.12.009

**Published:** 2023-02-20

**Authors:** Tiffany R. Hamilton, Arash A. Sabati, Todd T. Nowlen, Erik G. Ellsworth, Christopher L. Lindblade

**Affiliations:** Center for Heart Care, Phoenix Children's, Phoenix, Arizona

**Keywords:** D-transposition of the great arteries, Isolated right subclavian artery, Pulmonary hypertension, Anomalous origin, Fetal echocardiography

## Abstract

• Isolated RSCA from the PA is a rare finding in d-TGA.
• Atypical differential cyanosis in d-TGA may indicate abnormal aortic branching.
• Fetal imaging can provide evidence of an isolated RSCA.

## Graphical abstract

**Figure:**
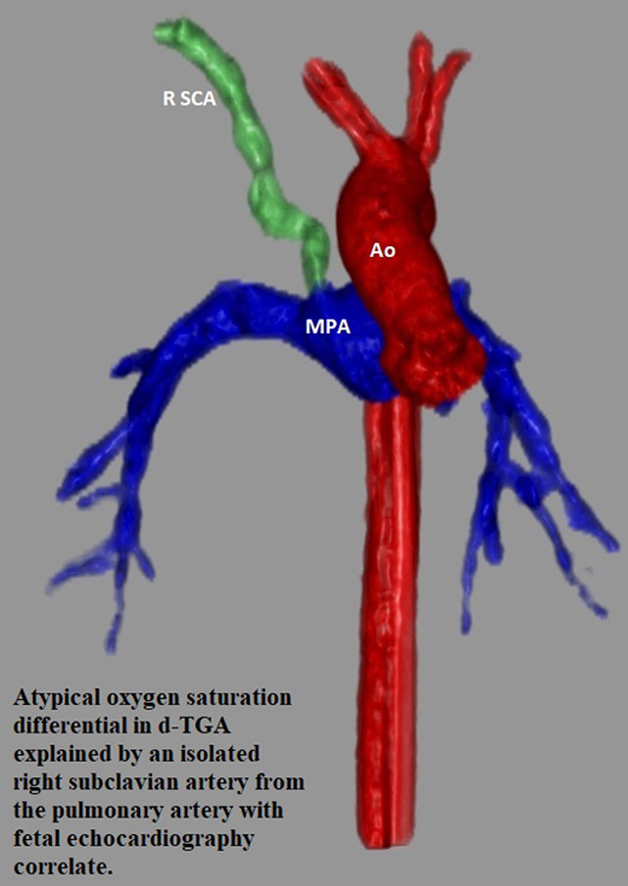

## Introduction

An isolated right subclavian artery (RSCA) from the pulmonary artery (PA) is a rare anomaly that has been associated with d-transposition of the great arteries (d-TGA) and may present with an atypical oxygen saturation differential upon infant delivery. This presentation can be further perplexing, with the known coexistence of a restricted atrial septum and a restrictive ductal arch in the fetus. Multimodality imaging is often required postdelivery to understand the anatomy. We present a case where this anomaly is discovered on retrospective review of fetal echocardiography.

## Case Presentation

Fetal echocardiography at 20 weeks gestation revealed the diagnosis of d-TGA with an intact ventricular septum. A follow-up echocardiogram at 31 weeks gestation also demonstrated evidence of a restrictive atrial septum (Video 1) and a restrictive ductal arch (Video 2). Immediately following an uncomplicated term delivery, the infant was transported to the intensive care unit, intubated for anticipated bedside balloon atrial septostomy, and placed on nitric oxide (iNO) for concern of pulmonary hypertension (PH) based on the fetal echocardiogram findings. Of note, the right upper extremity (RUE) oxygen saturations were consistently >90%, while those in the left upper extremity (LUE) and lower extremities (LE) were only 50%. The initial postnatal echocardiogram showed no ductal artery flow and only a small foramen ovale, so a prostaglandin infusion was initiated. Balloon atrial septostomy was then urgently performed 44 minutes after delivery, resulting in unrestrictive atrial level shunting and an initial increase in the LUE and LE oxygen saturations to 60%, while the RUE saturations remained >90%. Immediately following the balloon atrial septostomy, the infant became bradycardic, hypoxic, and hypotensive, requiring brief resuscitation, presumably secondary to a pulmonary hypertensive crisis.

Following initial resuscitation, the infant remained on 100% oxygen and iNO. Interestingly, the oxygen saturations in the RUE were consistently ≥95%, while those in the LUE and LE remained <65%. There was concern for inadequate mixing at the atrial level. However, a repeat echocardiogram demonstrated unrestrictive left-to-right atrial shunting, a small, bidirectional patent ductus arteriosus (PDA) measuring 2 mm in diameter, and no branching of the first branch off the aorta (Video 3). This echocardiogram suggested that the RSCA arose from the descending aorta near the arterial duct insertion. The bidirectional shunting at the PDA prompted further PH treatment with volume repletion, sedation, muscle relaxation, and inhaled iloprost. Interestingly, the O_2_ saturation differential between the UE and LUE/LE persisted. The patient did not tolerate iloprost, becoming hypotensive despite escalating dosing of epinephrine, and was subsequently discontinued. The umbilical artery blood gas revealed a PaO_2_ of only 20 mm Hg, and, due to persistent severe hypoxemia, the infant was placed on venoarterial extracorporeal membrane oxygenation (VA ECMO). There was subsequent improvement of the LUE and LE saturations to >90% with an umbilical artery catheter PaO_2_ of 80 mm Hg after cannulation. The RUE saturations remained >95% throughout this time.

### Differential Diagnosis

Given the severity of the atrial septal and ductal artery restriction in the setting of d-TGA, persistent PH was prenatally anticipated and presumed postnatally based on persistent hypoxemia and clinical history. Hemodynamics were not assessed by cardiac catheterization secondary to patient instability. However, this infant with d-TGA also presented with an unusual oxygen saturation differential, with low saturations in the LUE and LE and high saturations in the RUE. Initially, the high RUE saturation was falsely reassuring of adequate atrial-level mixing, while the decreased LUE and LE saturations were initially thought to be secondary to compromised systemic perfusion or possible equipment error. Once we confirmed the reliability of these saturations, we postulated that the higher saturation in the RUE might be secondary to unusual, differential streaming at the ductal level or perhaps from preferential flow from the arterial ECMO cannula into the brachiocephalic trunk. However, the saturation differential was present before and after atrial septostomy and ECMO cannulation. Repeat transthoracic echocardiography again demonstrated an abnormal course of the RSCA, although the exact origin was not entirely clear. Further advanced imaging was obtained once the infant was stabilized on VA ECMO.

### Investigations

**Figure 1:**
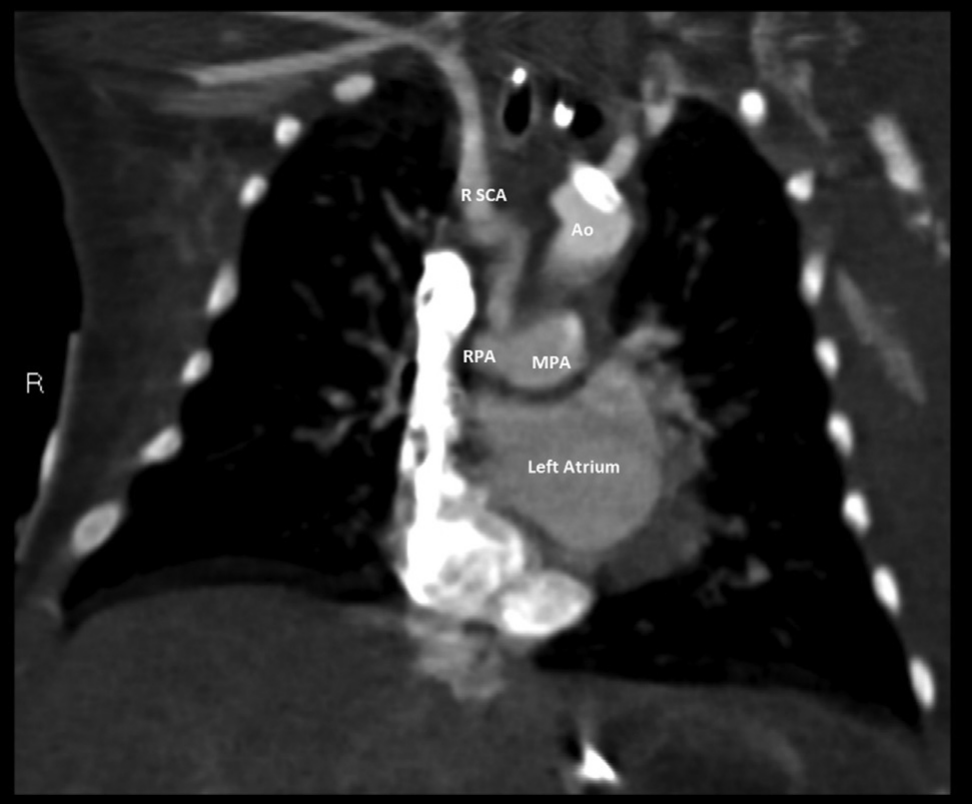
Multiplanar reformatted contrast-enhanced CCT demonstrates the RSCA arising from near the bifurcation of the PA (Philips Healthcare, 256 multislice; submillimeter axial slices with retrospective electrocardiogram gating, 40% phase).

**Figure 2:**
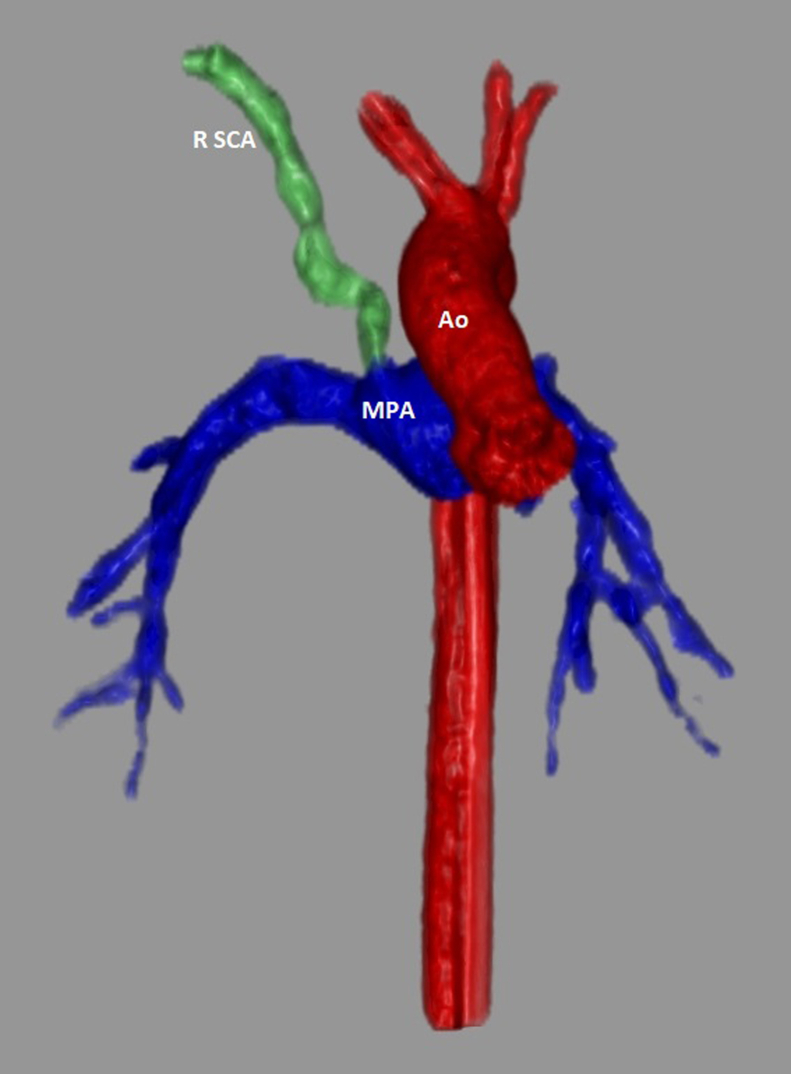
Three-dimensional volume rendering of the CCT demonstrates the RSCA (*green*) arising from the proximal right PA just beyond the main PA (MPA, *blue*). The aorta (Ao, *red*) and great vessels are well visualized.

A cardiac computed tomography scan (CCT) was performed while the infant was on VA ECMO that demonstrated an isolated RSCA arising from the PA, just beyond the bifurcation (Figures 1 and 2, Video 4).

### Management

**Figure 3:**
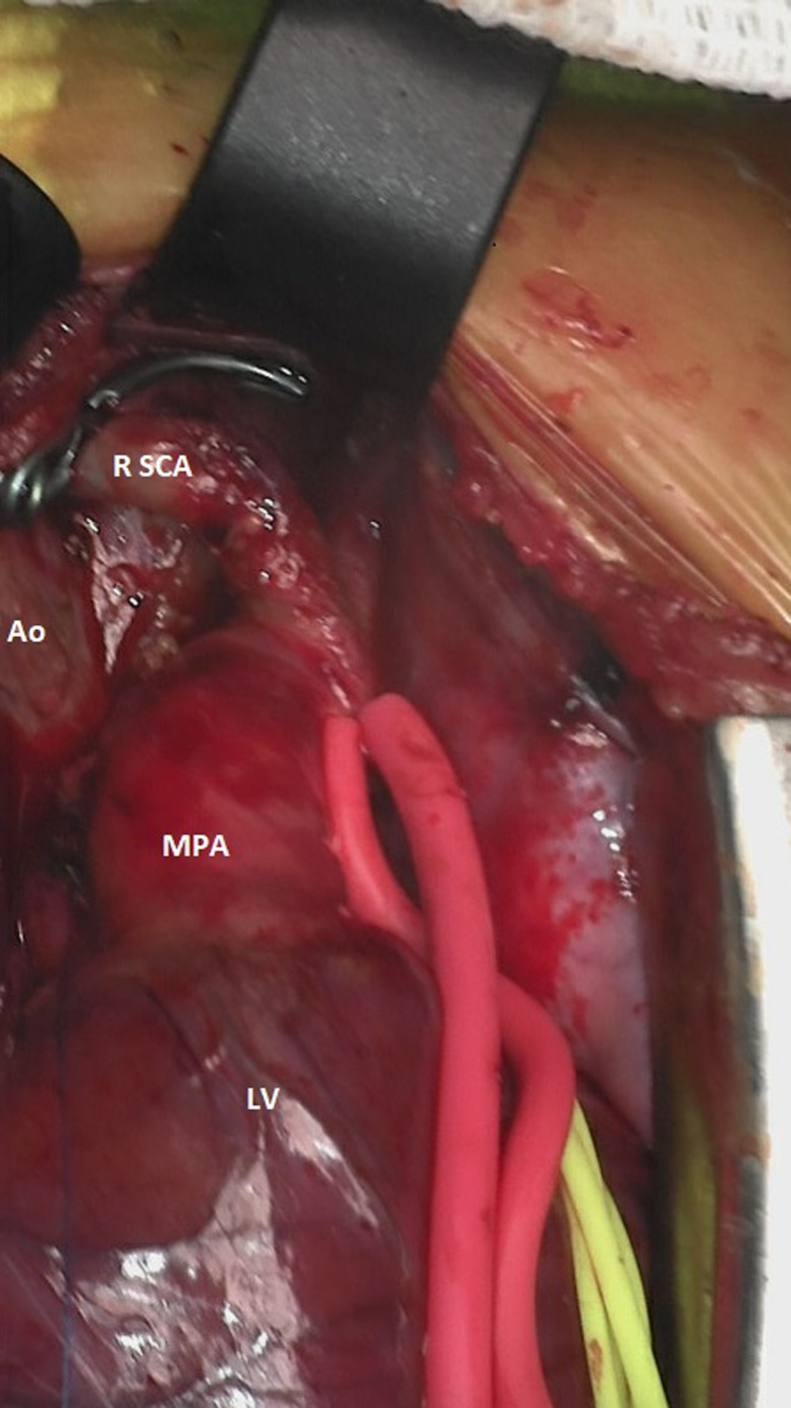
Intraoperative image with the anterior aorta (Ao) moved mostly out of the display toward the right and showing the main PA (MPA) with the RSCA arising from near the bifurcation and coursing to the right. *LV*, Left ventricle.

Once on full VA ECMO support, aggressive PH therapy was continued with iNO and reinitiation of inhaled iloprost. Hemodynamics and oxygenation improved, ECMO support was weaned, and the patient was decannulated after 72 hours of ECMO support while remaining on PH therapy. On day of life 9, the patient went to the operating room for an arterial switch procedure and transfer of the RSCA from the PA to the origin of the right common carotid artery (Figure 3). The patient had an uneventful postoperative course and was discharged home on sildenafil, which was weaned off at 1 month of age.

### Follow-up

The infant was discharged 3 weeks after surgery and remained on sildenafil for an additional month with subsequent resolution of PH. Follow-up echocardiogram demonstrated unobstructed flow at the RSCA anastomosis from the right common carotid artery and an excellent surgical result from the arterial switch operation. The patient is now 10 months old and reaching normal developmental milestones.

## Discussion

**Figure 4:**
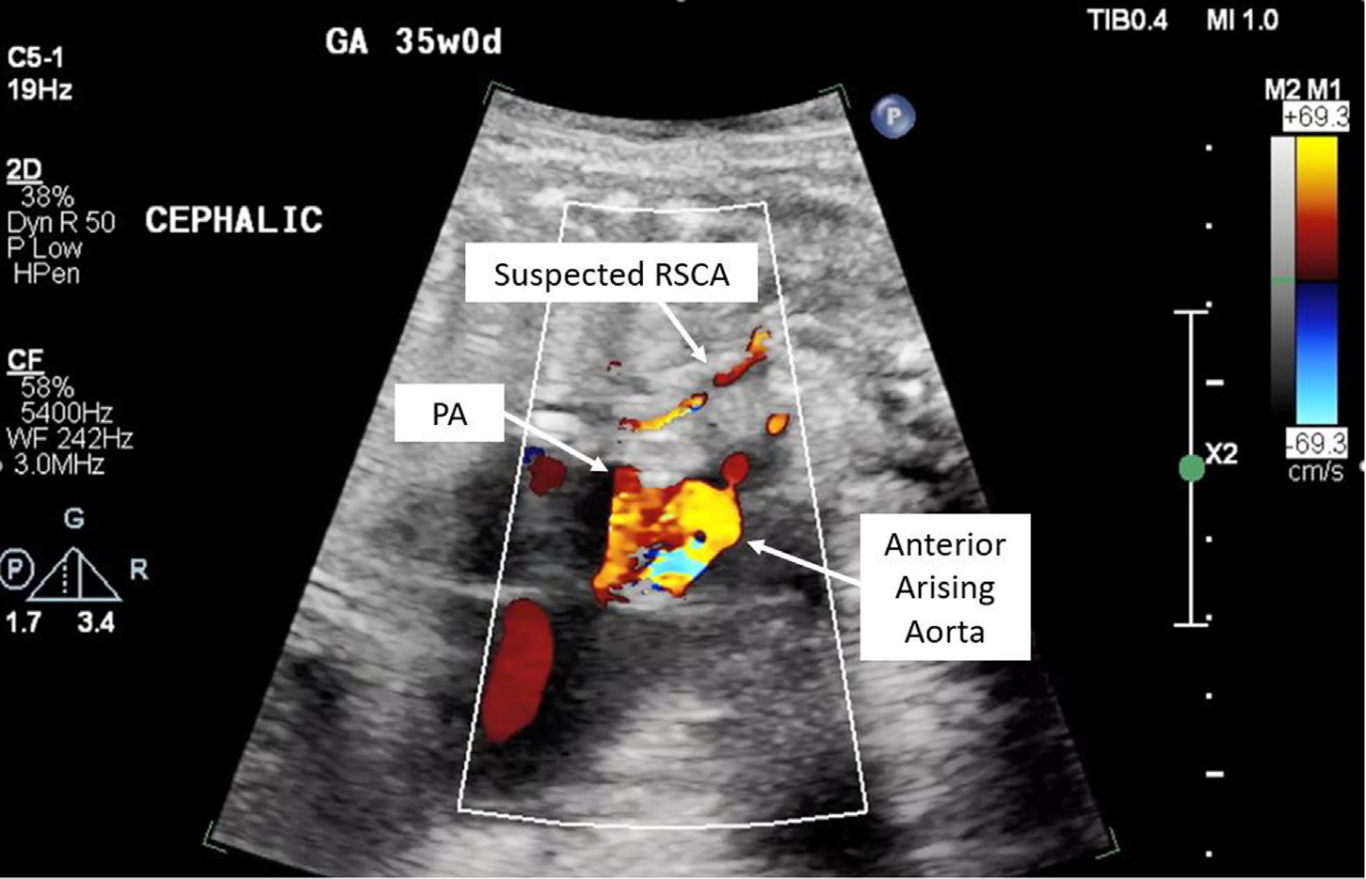
Two-dimensional fetal echo image with color flow Doppler, oblique sagittal orientation, demonstrates anteriorly arising aorta and the suspected RSCA appearing to arise from the PA.

This is a unique case of an infant with a prenatal diagnosis of d-TGA with intact ventricular septum, restrictive atrial septum, and ductal artery with an additional postnatal diagnosis of isolated RSCA arising from the PA. However, after retrospective correlation of the fetal echocardiogram with postnatal CCT imaging, fetal echo imaging suggests the diagnosis of the isolated RSCA arising from the PA on oblique sagittal color Doppler imaging (Video 5; Figure 4). The origin, course, and directionality of a pulsatile vessel in this view serves as a sonographic clue for this rare vascular anomaly. Further interrogation of this vessel by fetal echo may have provided a definitive prenatal diagnosis. This case demonstrates the possibility of prenatal identification.

The unusual oxygen saturation differential in our patient can be explained by this rare vascular anomaly, as fully saturated blood in the PA perfused the isolated RSCA, while mixed (at least partially desaturated) blood perfused the remainder of the aorta. This was even more pronounced in our patient due to the predominantly right-to-left shunting at the PDA. The saturation differential between the RUE and LUE/LE persisted even while on ECMO support, as there was still some degree of desaturated, prograde flow across the aortic valve. As this constellation of defects can lead to confusion and potentially adverse outcomes, there should be an increased index of suspicion in infants with a higher oxygen saturation in the RUE compared with the other extremities in d-TGA. Early recognition then provides the opportunity for appropriate medical management, surgical planning, and a successful postoperative outcome.

D-TGA can be associated with aortic arch anomalies in approximately 15% of cases, with the most common being coarctation of the aorta and right aortic arch.^1^ At this time, there are only 4 other reported cases of d-TGA with isolated RSCA arising from the PA. However, none of these cases provides fetal echo imaging of this rare vascular anomaly or in association with significant atrial and ductal artery restriction, which can further confound the clinical picture.^1^, ^2^, ^3^, ^4^ This case is unique as it provides a fetal echo correlate with postnatal CCT imaging of an isolated RSCA arising from the PA. Identification of an atypical pulsatile vessel arising from the PA such as seen here should alert the clinician to investigate further for this rare vascular anomaly that may occur with d-TGA.

## Conclusion

Isolated RSCA from the PA must be considered with atypical differential cyanosis in d-TGA infants particularly with PH. Identification of an isolated RSCA from the PA on fetal echocardiography is possible and should alert the clinician to consider this rare vascular anomaly prenatally.

## Ethics Statement

The authors declare that the work described has been carried out in accordance with The Code of Ethics of the World Medical Association (Declaration of Helsinki) for experiments involving humans.

## Consent Statement

The authors declare that since this was a non-interventional, retrospective, observational study utilizing de-identified data, informed consent was not required from the patient under an IRB exemption status.

## Funding Statement

The authors declare that this report did not receive any specific grant from funding agencies in the public, commercial, or not-for-profit sectors.

## Disclosure Statement

The authors report no conflict of interest.

## Supplementary Data

Video 1 Fetal echocardiogram, modified 4-chamber view, demonstrates a mildly thickened atrial septum *(arrow)* with decreased excursion of the foramenal flap.

Video 2 Fetal echocardiogram, sagittal view, demonstrates the aorta arising anteriorly with a qualitatively small ductal artery off the aortic arch.

Video 3 Two-dimensional transthoracic echocardiography, suprasternal notch sweep with color flow Doppler, demonstrates the first vessel off of the aorta does not branch. The aorta and brachiocephalic arteries are labeled *(arrows)*.

Video 4 Three-dimensional volume-rendered CCT cine-rotating image demonstrates the aorta *(red)* and the RSCA *(green)* arising from the proximal right PA *(blue)*.

Video 5 Two-dimensional fetal echocardiogram image with color flow Doppler, oblique sagittal orientation, demonstrates anteriorly arising aorta and the suspected RSCA appearing to arise from the PA. The right common carotid artery is labeled *(arrow)* for orientation.
